# Proportion of ALGBT adult Brazilians, sociodemographic characteristics, and self-reported violence

**DOI:** 10.1038/s41598-022-15103-y

**Published:** 2022-07-01

**Authors:** Giancarlo Spizzirri, Raí Álvares Eufrásio, Carmita Helena Najjar Abdo, Maria Cristina Pereira Lima

**Affiliations:** 1 Department of Psychiatry, Faculdade de Medicina FMUSP, Universidade de São Paulo, São Paulo, SP Brazil; 2 Department of Neurology, Psychology and Psychiatry, Botucatu Medical School, Universidade Estadual Paulista (UNESP), Botucatu, SP Brazil; 3 Independent researcher, São Paulo, SP Brazil

**Keywords:** Psychology, Human behaviour

## Abstract

Asexual, lesbian, gay, bisexual, and trans (ALGBT) individuals face worse life conditions and violence rates than their heterosexual cisgender counterparts. Brazil is often highlighted for having one of the highest rates of hate-related homicides against ALGBTs in the world. However, to date, Brazil’s ALGBT population has not been investigated with a representative sample, and basic information such as population size or sociodemographic characteristics are mostly based in non-systematic data. We aimed to assess the proportion of asexual, lesbian, gay, bisexual, trans and non-binary adults in Brazil, their sociodemographic characteristics, and self-reported violence rates. In 2018, a sample (n = 6000) of the Brazilian adult population answered a face-to-face survey assessing sociodemographic characteristics, gender identity, sexual orientation, and self-reported psychological, physical, verbal, and sexual violence. Among Brazilian adults, 12.04% are ALGBT: 5.76% asexual, 0.93% lesbian, 1.37% gay, 2.12% bisexual, 0.68 trans, and 1.18% non-binary. Compared to heterosexual cisgender men, most ALGBT individuals have worse socioeconomic indicators and higher rates of self-reported psychological and verbal violence. All ALGBT groups and heterosexual cisgender women reported sexual violence more often than heterosexual cisgender men. It was reported between 4 up to 25 times more often by heterosexual cisgender women and trans individuals, respectively. The rates of the other ALGBT groups sit among the two. Our findings provide evidence of the important size of the ALGBT Brazilian population, as well as their socioeconomic vulnerability, and concerning violence levels experienced by the group. Policy makers may refer to the present article in order to mitigate this population’s vulnerability and to better understand its sociodemographic characteristics.

## Introduction

Asexual, lesbian, gay, bisexual, and trans (ALGBT) individuals face worse life conditions and violence rates than their heterosexual cisgender counterparts. The group struggles with socioeconomic inequality^1^, stigma, and discrimination^2^. This has a negative effect on school and work, as well as on the access to health services^3–5^. As a consequence, ALGBT individuals have higher rates of physical and mental health issues^4,6–11^. Studies with representative samples estimate that the proportion of LGB people in the population sits around 3.5%^12–14^, whereas that of transgender and gender-diverse people varies between 0.1 and 2%^15^. Although public policies and programs strive at mitigating the hardship of ALGBT persons^13^, equity seems to be a long way ahead.

Violence also disproportionately affects ALGBT individuals since childhood^16–18^. According to the World Health Organization, violence is a complex phenomenon and may be defined as “the intentional use of physical force or power, threatened or actual, against oneself, another person, or against a group or community, that either results in or has a high likelihood of resulting in injury, death, psychological harm, maldevelopment or deprivation”^19^. Its nature may be categorized as physical, sexual, psychological, and deprivation or neglect. Violence motivated by hate against gender and/or sexuality diverse persons is reported not only in scientific papers^5,17,20^, but are often found in news outlets^21^. This is especially true for Brazil, one of the countries with the highest number of violent events leading to the death of ALGBT persons^22,23^.

By means of a face-to-face survey of a representative sample of Brazil’s adult population, this cross-sectional study sought to investigate the proportion of ALGBT adults in Brazil, describe their sociodemographic characteristics, and self-reported psychological, verbal, physical, and sexual violence. This study is written in the context when the decennial census conducted by the Brazilian Statistics Institute (IBGE) does not plan to collect data about gender diversity and sexual orientation in 2022^24^. The present study is, therefore, the first systematic assessment of the ALGBT population in the country.

## Method

Brazil is a large country, with 26 states (each with its own capital city and metropolitan area), and a federal district. The states are grouped in five geographical regions (North, Northeast, Midwest, Southeast and South). The population is distributed as follows: North 8%, Northeast 26%, Midwest 7%, Southeast 44%, and South 15%. Fifty-eight percent of Brazilians live in metropolitan areas, whereas 42% live in the countryside. The estimated adult population in December 2018 was 158,000,000 people^25^.

Between November and December 2018, a representative sample of the Brazilian adult population (18 years or older, n = 6000) was assessed by DataFolha Research Institute using a complex sampling method. The sample was stratified by Brazilian subregion, state, city, age group, gender perceived by the interviewer and level of education. In order for the sample to be representative of the Brazilian adult population, the following procedures were adopted: (i) the total number of participants to be interviewed in each geographic region was calculated considering the proportion of the Brazilian population living in that area; (ii) the same calculation process was then used for each state and cities; (iii) cities, neighborhoods and interview venues (squares, crossroads, avenues, business streets, etc.) were randomly picked. A hundred and twenty-nine cities were drawn from a total of 5561 cities, with a probability proportional to their populations. This method allows each city and the groups of cities to have demographic representation in the sample. Participants were randomly picked in the public venues.

Interviewers were instructed to collect data from a previously agreed number of people of both genders as perceived by the interviewer (male or female) and of all age groups. In order to ensure representativeness, after having collected data from a number of individuals, socioeconomic status and geographic region were also used to adjust sampling. Interviewers were trained regarding gender and sexual diversity and instructed to adopt a welcoming and impartial attitude towards the interviewees’ questions and answers.

Questions were divided into two sections: the first assessed sociodemographic characteristics (General Instrument, GI), whereas the second assessed specific aspects of one’s gender identity, sexual orientation, and reports of violence (“Specific instrument (SI)”), as described under section “Measures”. Individual interviews took, on average, 15 min.

The study was approved by the Ethics Committee of São Paulo State University (UNESP), Medical School, Botucatu Campus, Brazil (protocol number: 2903853). All methods were carried out following relevant guidelines and regulations. Informed consent was obtained from all subjects before the interviews. Participants did not receive financial incentive to take part in the study.

### Measures

#### General instrument (GI)

Participant’s sociodemographic characteristics were assessed with items regarding: geographic region where data was collected (southeast, south, northeast, central west, or north); urbanity (metropolitan area, or countryside); age in years; relationship status (in a relationship, or not in a relationship); level of education (up to high school, or higher education); economically active population (EAP) (EAP, not EAP); number of children; and social class (according to average family income) divided in 3 groups (A/B, above US$ 1380.00/month; C, between US$ 434.00 and US$ 760.00/month; or D/E, around US$ 182.00/month)^26^. US Dollar values considered the mean exchange rate in December 2018.

#### Specific instrument (SI)

Gender identity was assessed through three questions. Question (Q)1: Which of the following options best describes how you currently feel? (I feel I am a man; I feel I am a woman; I feel I am neither a man nor a woman). Q2: And what is the sex on your birth certificate? (male; female; undetermined). Q3: Which of these situations do you most closely relate to? (I was born male, but I have felt female since childhood; I was born female, but I have felt male since childhood; I was born male and I feel comfortable with my body; I was born female, and I feel comfortable with my body). Individuals that identified themselves with the gender assigned at birth were categorized as cisgender. Persons who identified with the binary gender opposite to their gender assigned at birth were categorized as transgender. Participants who identified with neither binary gender were categorized as non-binary.

Sexual orientation was assessed with Q4: Currently you feel attracted to, want to have sex or a relationship with, or fantasize about: (Only men; Only women; Men and women; Men and sometimes women; Women and sometimes men; I do not feel sexual attraction). Individuals were categorized as heterosexual when they reported only feeling attracted to the binary gender opposite to theirs. Women who reported only feeling attracted to other women were categorized as lesbian. Men who reported only feeling attracted to other men were categorized as gay. Individuals were categorized as bisexual when they reported feeling attracted to both binary genders. Participants who reported not feeling sexual attraction were categorized as asexual. These participants were further asked Q4a: Throughout your life, have you ever felt sexual attraction? (No, I have never felt sexual attraction; Yes, I have felt sexual attraction). With Q1, Q2, Q3, and Q4, the variable Group was created (categories: heterosexual cis man [HCM], heterosexual cis woman [HCW], lesbian, gay, bisexual man, bisexual woman, trans man, trans woman, non-binary, asexual man and asexual woman). Trans and non-binary individuals might also be subcategorized into different sexual orientations. However, this would render analyses unreliable due to lack or absence of cases in certain categories. For a detailed description of classification criteria, see Supplementary Table 1.

Q5 asked: Have you ever suffered: (a) Psychological violence, such as threats; (b) physical violence, such as slaps, kicks, punches, knife stabbing, etc.; (c) verbal violence, such as being cursed at or offended; (d) sexual violence, such as sexual abuse. For each type of violence, there were two answer options: Yes or No. For all questions in both GI and SI, the last answer options were: Do not know/do not understand the question; Refuse to answer.

### Statistical analysis

All analyses considered a complex sample plan, which informed that data was stratified and cases were weighted by subregion, state, city, age group, gender perceived by the interviewer and level of education. The estimation method was WR (sampling with replacement) corrected for sampling from a finite population when estimating the variance under the complex sampling design.

Logistic regression models analyzed univariate associations between sociodemographic factors and variable Group. Factors significantly associated with Group were included in a multivariate logistic regression model, followed by simple contrast comparisons (reference category = HCM).

Four binary logistic regression models were fit to assess the predictive relationship between Group and each type of violence, adjusted for sociodemographic variables related to violence (social class, education level, Brazilian subregion, urbanity, and age)^27^. For the analysis of sexual violence, a version of the variable Group with fewer categories was used (categories: HCM, HCW, lesbian, gay, bisexual man, bisexual woman, trans individuals, non-binary, asexual individuals). This was necessary due to lack or absence of cases in certain subcategories.

Where applicable, p-values were adjusted for multiple comparisons with the Bonferroni correction. P-values were 2-tailed and statistical significance was considered when p ≤ 0.05. All analyses were performed using SPSS version 22.0.

## Results

All n reported herein are not design-adjusted, whereas all percentages and confidence intervals (CI) are adjusted. From the sample of 6000, a total of 142 individuals (2.44%) were not categorized in the Group variable for lack of responses. Therefore, the sample used in analyses was 5858, 270 of whom were categorized as LGB (4.42%): 55 lesbian (0.93%), 83 gay (1.37%), 43 bisexual men (0.70%), and 89 bisexual women (1.42%), 325 asexual (5.76%): 22 men (0.37%) and 303 women (5.39%). 111 people were categorized into gender-diversity groups (1.87%): 20 trans men (0.34%), 20 trans women (0.34%), and 71 non-binary persons (1.18%). 706 people (12.04%, CI 95% = 10.05–14.57%) were categorized as ALGBT. For details see Supplementary Table 2.

### Sociodemographic characteristics

HCM are more likely to be in: (a) higher social classes than HCW, lesbian, and asexual women, (b) EAP than HCW, bisexual women, trans men, and asexual men and women, (c) metropolitan areas than HCW, and bisexual women, (d) a relationship than lesbian, gay, bisexual men and women, trans women, asexual men and women. HCM are less likely to be in higher education than HCW, lesbian, gay and bisexual men and women. On average, HCM: (a) are older than HCW, lesbian, bisexual women, trans men and women, and younger than asexual men and women, (b) have more children than gay, and asexual men, and less than HCW and asexual women. No significant effect was found for variable Subregion. Sample size, percentages, and CI95% of each subgroup are shown in Supplementary Table 2. Odds ratios and CI95% for each pairwise comparison are shown in Table 1.

**Table 1:** Predictive effect of group on sociodemographic characteristics.

OR of belonging to a category or having a different mean						
Variable	Category	Group	OR	CI 95%		*P*
Social class	A/B vs. D/E	Heterosexual cis woman	0.52	0.44	0.61	< 0.001
		Lesbian	0.28	0.13	0.64	0.002
		Asexual woman	0.33	0.21	0.49	< 0.001
		Heterosexual cis man	1.00			
	C vs. D/E	Heterosexual cis woman	0.80	0.70	0.92	0.001
		Asexual woman	0.59	0.44	0.79	< 0.001
		Heterosexual cis man	1.00			
Urbanity	Countryside vs. metropolitan area	Heterosexual cis woman	1.11	1.05	1.18	< 0.001
		Bisexual woman	1.93	1.28	2.93	0.002
		Heterosexual cis man	1.00			
Relationship status	Not in a relationship vs. in a relationship	Lesbian	3.45	1.64	7.27	0.001
		Gay	2.32	1.47	3.66	< 0.001
		Bisexual man	3.94	2.01	7.73	< 0.001
		Bisexual woman	3.63	1.99	6.64	< 0.001
		Trans woman	2.77	1.00	7.63	0.05
		Non-binary	2.33	1.39	3.93	0.001
		Asexual man	3.03	1.20	7.65	0.02
		Asexual woman	4.31	3.23	5.75	< 0.001
		Heterosexual cis man	1.00			
Education	Up to high school vs. higher education	Heterosexual cis woman	0.55	0.51	0.60	< 0.001
		Lesbian	0.37	0.20	0.67	0.001
		Gay	0.36	0.23	0.56	< 0.001
		Bisexual man	0.35	0.18	0.67	0.002
		Bisexual woman	0.43	0.28	0.66	< 0.001
		Heterosexual cis man	1.00			
EAP	EAP vs. not EAP	Heterosexual cis woman	0.50	0.45	0.56	< 0.001
		Bisexual woman	0.45	0.29	0.69	< 0.001
		Trans man	0.23	0.10	0.56	0.001
		Asexual man	0.40	0.19	0.85	0.02
		Asexual woman	0.42	0.31	0.57	< 0.001
		Heterosexual cis man	1.00			
Age	Change in 1 year	Heterosexual cis woman	0.98	0.98	0.99	< 0.001
		Lesbian	0.97	0.94	0.99	0.01
		Bisexual woman	0.93	0.91	0.96	0.006
		Trans man	0.95	0.92	0.98	0.001
		Trans woman	0.97	0.94	1.00	0.04
		Asexual man	1.06	1.03	1.09	< 0.001
		Asexual woman	1.06	1.05	1.07	< 0.001
		Heterosexual cis man	1.00			
Number of children	Change in 1 unit	Heterosexual cis woman	1.21	1.15	1.27	< 0.001
		Gay	0.65	0.48	0.89	0.006
		Asexual man	0.70	0.53	0.92	0.01
		Asexual woman	1.17	1.07	1.29	0.001
		Heterosexual cis man	1.00			

*OR* odds ratio, *CI* confidence interval, *EAP* economically active population. Only statistically significant comparisons are reported.

### Asexual individuals

Women make up most of the sample of asexual individuals (n = 303, 93.5%). Asexual men are equally divided between those who have felt sexual attraction throughout life (n = 11, 50.6%) and those who have never (n = 11, 49.4%). Asexual women, however, report having felt sexual attraction before in 82.3% of cases (n = 246). Asexual individuals who have never felt sexual attraction make up 1.1% of the sample (n = 62), whereas those who have felt represent 4.5% (n = 257). In general, those who have felt sexual attraction before are on average older than those who have never, however, such difference is larger for men (mean age difference = 23.6 years) than for women (mean age difference = 2.6 years) (see Table 2). Due to the small size of some subgroups, this analysis with age is preliminary.

**Table 2:** Percent number and mean age of Asexual men and women for each option of Q4a.

Q4a. Throughout your life, have you ever felt sexual attraction?	Asexual men (n = 22, 6.5%)		Asexual women (n = 303, 93.5%)	
	n (%)	Mean age	n (%)	Mean age
No, I have never felt sexual attraction	11 (50.6%)	42.4	51 (17.7%)	57.7
Yes, I have felt sexual attraction	11 (49.4%)	65.9	246 (82.3%)	60.3

Percentages and means were design-adjusted.

### Violence

Supplementary Table 3 summarizes the association between variable Group and each type of violence. The binary logistic regression analyses showed that Group significantly predicted the outcome of psychological, physical, verbal, and sexual violence, controlled for social class, education level, urbanity, Brazilian subregion, and age. ORs and CI95% of pairwise comparisons against HCM are shown in Table 3. Bisexual women, trans men and women, non-binary persons, and asexual women are more likely than HCM to report psychological violence. HCW are less likely than HCM to report physical violence. HCW and gay are less likely than HCM to report verbal violence, while non-binary people and asexual women are more likely. All groups are more likely than HCM to report sexual violence.

**Table 3:** Predictive effect of Group on psychological, physical, verbal, or sexual violence.

OR of reporting psychological, physical, verbal, or sexual violence				
Violence type	OR	CI95%		*p*
**Psychological**				
Bisexual woman	2.09	1.35	3.24	< 0.001
Trans man	3.81	1.61	9.02	0.002
Trans woman	3.15	1.31	7.57	0.01
Non-binary	1.83	1.12	3.01	0.02
Asexual woman	1.45	1.11	1.89	0.007
Heterosexual cis man	1.00			
**Physical**				
Heterosexual cis woman	0.64	0.56	0.72	< 0.001
Heterosexual cis man	1.00			
**Verbal**				
Heterosexual cis woman	0.85	0.77	0.95	0.004
Gay	0.67	0.45	1.00	0.05
Non-binary	2.18	1.29	3.67	0.003
Asexual woman	1.28	1.00	1.62	0.05
Heterosexual cis man	1.00			
**Sexual**				
Heterosexual cis woman	4.12	3.04	5.57	< 0.001
Lesbian	5.95	2.48	14.24	< 0.001
Gay	5.46	2.53	11.77	< 0.001
Bisexual man	6.67	2.72	16.37	< 0.001
Bisexual woman	12.94	7.35	22.79	< 0.001
Trans	25.48	13.02	49.87	< 0.001
Non-binary	15.17	8.14	28.27	< 0.001
Asexual	4.82	3.02	7.69	< 0.001
Heterosexual cis man	1.00			

*OR* odds ratio, *CI* confidence interval. Only statistically significant comparisons are reported.

## Discussion

This is the first study to assess the proportion of ALGBT people in a Latin American country with a representative sample. From the Brazilian adult population (estimated at 158,000,000 at the time of data collection^25^), 12% or approximately 19 million individuals are ALGBT and are homogeneously distributed throughout the country. We found that 4.42% of Brazilians are LGB. Studies with representative samples show that around 3.5% of participants self-identify as LGB in US and Australia^12,14^, and 2.2% in the UK. In New Zealand, 3.9% of women identify as lesbian or bisexual, 5% of men identity as gay or bisexual, and 1.3% as bicurious. Considering the stigma around being ALGBT, and since our data were collected in person, the percentages found herein may be underestimated. Because of marginalization, ALGBTs are a hard-to-reach population, whose sampling is either biased in nonrandom methods (i.e., snowball sampling) or unspecific in common random sampling methods. The aforementioned studies with representative samples have samples larger than ours. However, they collected data remotely, whereas we surveyed participants face-to-face. We believe this method to be better in the case of our country, since the number of Brazilians with limited access to the internet and telephone is still significant.

Similar to previous studies^12,14^, we found that the group of bisexual women (1.42%) is larger than that of bisexual men (0.70%), and more men report feeling sexual attraction to the same gender only (1.37% *vs* 0.93% of women). An online study with nonrepresentative samples of 28 countries observed that the proportions of LGB persons vary between 7 and 15%^28^. A systematic review estimating the proportion of men who have sex with men in low and middle income countries found percentages between 6 and 20%^29^. A British study showed that 4.9% of women reported having had sex with another woman^30^.

Asexuality may be generally understood as the absence of sexual attraction^31,32^. In our study, 1.1% of participants reported not having felt sexual attraction before, whereas 4.5% do not feel sexual attraction, but have felt before. In 2004, the first study on the subject found that approximately 1% of British residents reported never having felt sexually attracted to anyone^33^. Estimates between 0.4 and 0.9% were observed in other studies^34–36^. However, a Finnish study showed that 4.8% (1.5% men, and 3.3% women) reported experiencing no sexual attraction in the 12 months previous to data collection^37^. The wide variation of estimates may be due to differences in the method to assess asexuality. Proportions of individuals categorized as asexual seem to be higher when the criterion is current absence of sexual attraction, when compared to long-lasting absence (such as life-long absence). Many factors may influence sexual attraction, which is variable throughout individuals’ lives. Whether solely current or long-lasting, asexuality should be considered in its full diversity. Significantly more women than men report absence of sexual attraction in the present study, and asexual women are older than asexual men, which is also found in other studies^33,37^. In a study in Britain, however, asexuality was not associated with gender or age^34^. The proportion of those who have never felt sexual attraction among asexual men is higher than among asexual women. However, due to the small sample of asexual men (n = 22), this finding is preliminary.

Supporting previous findings^4^, we show that LGB people and HCW have a higher education level than HCM. Nevertheless, HCM show better socioeconomic indicators than most of these groups. The reasons behind the higher education levels among sexual minorities remain unknown, but the result may indicate an effort the group makes to compensate for the exclusion society imposes over them. If so, such effort does not seem to fairly translate into better socioeconomical indicators. Along with this, HC and asexual women are less likely to be in a relationship and have more children on average. According to IBGE, women in our country study and work more than men, earn less, and are responsible for most of the children’s care work^38^. This brings to gender inequality discussions^39^ not only the matter of income disparity, but also that care work (usually performed by women) is far from being fairly compensated.

We found that LGB people are less likely to be in a relationship and have less children than HCM on average. Other studies also observed that LGB people were more likely to never get married^4,40^. Brazil’s Supreme Federal Court has made same-sex civil unions legal since 2011. This decision is very recent and laws discussing adoption by LGBT families still face great opposition in the country^41^. Although society has increasingly recognized family structures that do not come from a heterosexual couple^42^, sexual minorities still face challenges while trying to start a relationship or a family.

In our study, 1.87% of participants are trans or non-binary, which is within the range previously found in a review study^15^, and is discussed more thoroughly in another article^43^. We found that trans women and non-binary persons are less likely to be in a relationship than HCM, a difference not observed in the US for trans individuals in a 2017 study^44^, yet found more recently, in 2020^45^. Trans individuals also face relationship challenges experienced by LGBs with the addition of being even more marginalized by society. Similar to previously shown^44,46^, we found that trans men are less likely to be part of the EAP than HCM. Trans people also showed a lower mean age than HCM, which corroborates past research^47,48^. Although no evidence has been produced, it is believed that life expectancy of trans Brazilians is around 35 years. Violence should explain a good part of it, since Brazil is the country with the highest rates of homicide against trans individuals^49^. Limited access to formal employment and to quality healthcare are other probable factors influencing the group’s life expectancy.

Violence was generally reported less by HCM than other groups. The exception being physical violence, which was reported less by HCW, and verbal violence, less frequent among gay and HCW. A Brazilian study analyzing notifications of violence against LGBT persons between 2015 and 2017 (24,564 notifications) found that 46.6% mention that the victim’s gender is trans, and 57.6% mention that the victim’s sexual orientation is lesbian or gay. It also shows that in 66.2% of cases the probable perpetrator is male^50^. Our question about violence did not specify motivations of hate against ALGBT persons, race/ethnicity, health problems, or of other nature. However, our findings indicates that violence disproportionately affects those who are not the “norm” (i.e., HCM).

Sexual violence is a generic term describing any form of unconsented sexual act or behavior, including: sexual abuse, rape, sexual assault, sexual coercion, among others^19^. For the victims, it impacts several areas of life, and disturbs both physical and mental health^51^. If, on the one hand, one could hypothesize important differences among the groups assessed in the present study, on the other hand, the magnitude of such differences for sexual violence are unmistakably the most concerning. When compared to HCM, this type of violence was reported 4 up to 25 times more by HCW and trans individuals, respectively. US studies also showed that LGB people experienced higher rates of sexual violence than their cisgender counterparts^52,53^. A systematic review about violence motivated by perception of sexual orientation and gender identity showed that rates of sexual violence vary from 2.1 to 9.7% for LGB people, and 7–49.1% for the trans population^54^. The proportions found in the present study are similar to these. The 2015 Asexual Community Census showed that 43.5% of asexual participants reported sexual violence^55^. HCW in our study also reported sexual violence much more often than HCM, which has been found before^51,56^. We should consider, however, that sexual violence may be perceived differently among groups, and that some may feel less comfortable reporting it.

Since this is a cross-sectional study, fluidity of gender identity and sexual attraction were not captured. We did not have specific questions about hate-motivated violence, which would shed yet more light into violence against the ALGBT population in Brazil. Race/ethnicity information was also not collected, due to the high variability in the perception of race in the country. Even though our question about sex included the option “undetermined”, we did not capture the group of intersex people. In order to evaluate sexual attraction of trans and non-binary individuals, a sample size larger than the one in this study will be necessary. The present study evaluates sexual attraction, and assesses gender identity indirectly. An extra question would be necessary to find out whether individuals would actually identify with the category they were assigned herein.

Brazil currently has no estimate of the size of its ALGBT population or whether the group is located in specific areas of the country. Socioeconomic and violence indicators available are concerning, yet are not result of systematic investigation. We found that gender and sexuality diverse people are homogenously located in all 5 Brazilian subregions, and represent at least 12% of the adult population. The poor socioeconomic indicators and high rates of violence among ALGBT persons found herein are of great concern, and provides yet more evidence of the inequality and vulnerability faced by the group. Countries where LGBT rights are respected also have better human development indices (HDI)^57^. The findings of the present study may help with the conception of public policies aimed at improving equity, which should be accompanied by a general improvement of human development in the country. This is especially important now that Brazil experiences worsening life conditions, having lost 5 positions in the world’s HDI ranking^58^.

## Supplementary Information

Supplementary Information.

## Data Availability

The dataset used and analyzed in the present study is available from the corresponding author upon reasonable request.
