# Supplementary material for: Proportion of ALGBT adult Brazilians, sociodemographic characteristics, and self-reported violence
Source: Sci Rep. 2022 Jul 1;12:11176. doi: 10.1038/s41598-022-15103-y (PMC9249838; doi:10.1038/s41598-022-15103-y)
Supplement: Supplementary file 1 — Supplementary Information. [file 41598_2022_15103_MOESM1_ESM.docx]

**Proportion of ALGBT adult Brazilians, sociodemographic characteristics, and self-reported violence**

# Supplementary material

Supplementary table 1. Group categorization (heterosexual cisgender man/woman, lesbian, gay, bisexual man/woman, transgender man/woman, non-binary gender, asexual man/woman) following the association between answers to Specific Instrument’s Q1, Q2, Q3, and Q4.

| **Group category** | **Q1: Which of the following best describes how you currently feel?** | **Q2: And what sex is registered on your birth certificate?** | **Q3: Which of these situations best describes you?** | **Q4: Currently you feel attracted to, want to have sex or a relationship with, or fantasize about** | **n** |
| --- | --- | --- | --- | --- | --- |
| Heterosexual cisgender man | I feel I am a man | Male | I was born man and I am comfortable with my body | Only women | 2548 |
|  | Do not know or Refuses to answer | Male | I was born man and I am comfortable with my body | Only women | 6 |
|  | I feel I am a man | Male | Do not know or Refuses to answer | Only women | 35 |
| **Total** | | | | | **2589** |
| Heterosexual cisgender woman | I feel I am a woman | Female | I was born woman and I feel comfortable with my body | Only men | 2535 |
|  | Do not know or Refuses to answer | Female | I was born woman and I feel comfortable with my body | Only men | 11 |
|  | I feel I am a woman | Female | Do not know or Refuses to answer | Only men | 17 |
| **Total** | | | | | **2563** |
| Lesbian | I feel I am a woman | Female | I was born woman and I feel comfortable with my body | Only Women | 51 |
|  | I feel I am a woman | Female | Do not know or Refuses to answer | Only Women | 4 |
| **Total** | | | | | **55** |
| Gay | I feel I am a man | Male | I was born man and I am comfortable with my body | Only man | 82 |
|  | Do not know or Refuses to answer | Male | I was born man and I am comfortable with my body | Only man | 1 |
| **Total** | | | | | **83** |
| Bisexual man | I feel I am a man | Male | I was born man and I am comfortable with my body | Both | 42 |
|  | I feel I am a man | Male | Do not know or Refuses to answer | Both | 1 |
| **Total** | | | | | **43** |
| Bisexual woman | I feel I am a woman | Female | I was born woman and I feel comfortable with my body | Only women | 86 |
|  | I feel I am a woman | Female | Do not know or Refuses to answer | Only women | 3 |
| **Total** | | | | | **89** |
| Transgender Man | I feel I am a man | Female | I was born female but I have felt male since childhood | Only men | 5 |
|  |  |  |  | Only women | 8 |
|  |  |  |  | Both | 1 |
|  |  |  |  | I do not feel sexual attraction | 2 |
|  | I feel I am a man | Female | I was born man and I am comfortable with my body | Only women | 2 |
|  | I feel neither a man nor a woman | Female | I was born female but I have felt male since childhood | Only women | 1 |
|  |  |  |  | Both | 1 |
|  | Do not know or Refuses to answer | Female | I was born female but I have felt male since childhood |  | 0 |
| **Total** | | | | | **20** |
| Transgender Woman | I feel I am a woman | Male | I was born male but I have felt female since childhood | Only men | 6 |
|  |  |  |  | Only women | 1 |
|  |  |  |  | Both | 1 |
|  | I feel neither a man nor a woman | Male | I was born male but I have felt female since childhood | Only men | 7 |
|  |  |  |  | Only women | 1 |
|  |  |  |  | Both | 2 |
|  |  |  |  | I do not feel sexual attraction | 1 |
|  | Do not know or Refuses to answer | Male | I was born male but I have felt female since childhood | Both | 1 |
| **Total** | | | | | **20** |
| Non-Binary | I feel neither a man nor a woman | Male | I was born man and I feel comfortable with my body | Only men | 9 |
|  |  |  |  | Only women | 14 |
|  |  |  |  | Both | 7 |
|  |  |  |  | I do not feel sexual attraction | 2 |
|  | I feel neither a man nor a woman | Male | Do not know or Refuses to answer | Both | 1 |
|  | I feel neither a man nor a woman | Female | I was born woman and I feel comfortable with my body | Only men | 21 |
|  |  |  |  | Only women | 1 |
|  |  |  |  | Both | 2 |
|  |  |  |  | I do not feel sexual attraction | 10 |
|  |  |  |  | Do not know or Refuses to answer | 2 |
|  | I feel neither a man nor a woman | Female | Do not know or Refuses to answer | Only men | 1 |
|  |  |  |  | I do not feel sexual attraction | 1 |
| **Total** | | | | | **71** |
| Asexual man | I feel I am a man | Male | I was born man and I am comfortable with my body | I do not feel sexual attraction | 22 |
| **Total** | | | | | **22** |
| Asexual woman | I feel I am a woman | Female | I was born woman and I feel comfortable with my body | I do not feel sexual attraction | 302 |
|  | I feel I am a woman | Female | Do not know or Refuses to answer | I do not feel sexual attraction | 1 |
| **Total** | | | | | **303** |
| **Grand total** | | | | | **5858** |

Supplementary table 2. Sociodemographic characteristics of each category of variable Group.

| **Variable** | ***p*** | **Group (n = 5858)** | |  |  |  |  |  |  |  |
| --- | --- | --- | --- | --- | --- | --- | --- | --- | --- | --- |
|  |  | **Heterosexual cis man, n = 2589 (42.5%, CI 95% = 42.01 to 43)** | | | **Heterosexual cis woman, n = 2563 (43.02%, CI 95% = 42.3 to 43.73)** | | | **Lesbian, n = 55 (0.93%, CI 95% = 0.72 to 1.21)** | | |
|  |  | **n (%)** | **CI 95%** | | **n (%)** | **CI 95%** | | **n (%)** | **CI 95%** | |
| **Social class** | <.001 |  |  |  |  |  |  |  |  |  |
| **D/E** |  | 540 (23.1) | 21.6 | 24.7 | 714 (30.2) | 28.6 | 31.8 | 13 (26.9) | 16.6 | 40.4 |
| **C** |  | 1170 (48.4) | 46.6 | 50.2 | 1198 (48.7) | 46.8 | 50.5 | 31 (56.8) | 43.6 | 69.0 |
| **A/B** |  | 879 (28.5) | 27.1 | 29.9 | 651 (21.2) | 19.9 | 22.4 | 11 (16.3) | 9.6 | 26.3 |
| **Urbanity** | <.001 |  |  |  |  |  |  |  |  |  |
| **Countryside** |  | 646 (23.2) | 22.7 | 23.8 | 659 (23.9) | 23.1 | 24.6 | 18 (31.5) | 20.7 | 44.7 |
| **Metropolitan area** |  | 1943 (76.8) | 76.2 | 77.3 | 1904 (76.1) | 75.4 | 76.9 | 37 (68.5) | 55.3 | 79.3 |
| **Relationship status** | <.001 |  |  |  |  |  |  |  |  |  |
| **Not in a relationship** |  | 1201 (46.8) | 45 | 48.5 | 1257 (49.4) | 47.6 | 51.3 | 45 (81.4) | 69 | 89.6 |
| **In a Relationship** |  | 1388 (53.2) | 51.5 | 55 | 1306 (50.6) | 48.7 | 52.4 | 10 (18.6) | 10.4 | 31 |
| **Subregion** | <.07 |  |  |  |  |  |  |  |  |  |
| **Southeast** |  | 1100 (43.3) | 42.7 | 43.9 | 1063 (42.4) | 41.6 | 43.3 | 23 (44.5) | 32.1 | 57.7 |
| **South** |  | 391 (14.6) | 14.2 | 15 | 412 (15.3) | 14.8 | 15.9 | 8 (13.5) | 7.2 | 24.1 |
| **Northeast** |  | 673 (26.2) | 25.7 | 26.7 | 664 (26.3) | 25.6 | 27 | 7 (13.1) | 6.6 | 24.4 |
| **Central west** |  | 214 (7.7) | 7.4 | 8 | 226 (8.2) | 7.8 | 8.6 | 7 (10.3) | 4.9 | 20.5 |
| **North** |  | 211 (8.2) | 7.8 | 8.5 | 198 (7.8) | 7.3 | 8.2 | 10 (18.5) | 10 | 31.8 |
| **Education** | <.001 |  |  |  |  |  |  |  |  |  |
| **Up to high school** |  | 2058 (81.6) | 81.1 | 82.1 | 1970 (78.7) | 78.1 | 79.3 | 36 (67.6) | 54.7 | 78.2 |
| **Higher education** |  | 531 (18.4) | 17.9 | 18.9 | 593 (21.3) | 20.7 | 21.9 | 19 (32.4) | 21.8 | 45.3 |
| **Belonging to EAP** | <.001 | 1862 (71.4) | 69.8 | 73 | 1472 (56.9) | 55.1 | 58.7 | 39 (70.7) | 57.9 | 80.9 |
| **Mean age** | <.001 | 42.2 | 42.0 | 42.5 | 40.9 | 40.6 | 41.3 | 32.8 | 28.9 | 36.7 |
| **Mean number of children** | <.001 | 1.51 | 1.47 | 1.55 | 1.70 | 1.65 | 1.74 | 0.84 | 0.50 | 1.19 |
| **Variable** | ***p*** | **Group (n = 5858)** | | **(Continued).** | |  |  |  |  |  |
|  |  | **Gay, n = 83 (1.37%, CI 95% = 1.13 to 1.65)** | | | **Bisexual man, n = 43 (0.7%, CI 95% = 0.53 to 0.94)** | | | **Bisexual woman, n = 89 (1.42%, CI 95% = 1.17 to 1.72)** | | |
|  |  | **n (%)** | **CI 95%** | | **n (%)** | **CI 95%** | | **n (%)** | **CI 95%** | |
| **Social class** | <.001 |  |  |  |  |  |  |  |  |  |
| **D/E** |  | 11 (15) | 9.2 | 23.6 | 8 (19.7) | 10.6 | 33.6 | 13 (17.1) | 10.4 | 26.9 |
| **C** |  | 40 (50.8) | 41.3 | 60.2 | 23 (58) | 43.3 | 71.3 | 33 (42.6) | 33.1 | 52.7 |
| **A/B** |  | 32 (34.2) | 26.2 | 43.2 | 12 (22.3) | 12.8 | 36.1 | 43 (40.3) | 31.5 | 49.7 |
| **Urbanity** | <.001 |  |  |  |  |  |  |  |  |  |
| **Countryside** |  | 23 (26.4) | 18.7 | 35.9 | 17 (36.5) | 24.0 | 51.3 | 40 (40.8) | 31.8 | 50.5 |
| **Metropolitan area** |  | 60 (73.6) | 64.1 | 81.3 | 26 (63.5) | 48.7 | 76.0 | 49 (59.2) | 49.5 | 68.2 |
| **Relationship status** | <.001 |  |  |  |  |  |  |  |  |  |
| **Not in a relationship** |  | 64 (76) | 66.2 | 83.7 | 35 (81.2) | 66.4 | 90.4 | 76 (84.5) | 75.4 | 90.6 |
| **In a Relationship** |  | 19 (24) | 16.3 | 33.8 | 8 (18.8) | 9.6 | 33.6 | 13 (15.5) | 9.4 | 24.6 |
| **Subregion** | <.07 |  |  |  |  |  |  |  |  |  |
| **Southeast** |  | 37 (44) | 35 | 53.5 | 21 (48.9) | 34.8 | 63.2 | 49 (56.7) | 47 | 65.8 |
| **South** |  | 19 (21.4) | 14.3 | 30.7 | 5 (12.8) | 6 | 25.1 | 16 (14.8) | 9.5 | 22.2 |
| **Northeast** |  | 17 (21.9) | 14.7 | 31.2 | 7 (16.8) | 8.2 | 31.3 | 12 (14.7) | 9.2 | 22.5 |
| **Central west** |  | 6 (7.9) | 4 | 14.9 | 4 (8) | 2.9 | 20.1 | 1 (1) | 0.1 | 6.9 |
| **North** |  | 4 (4.8) | 2.1 | 10.8 | 6 (13.5) | 6.1 | 27.3 | 11 (12.9) | 7.6 | 20.9 |
| **Education** | <.001 |  |  |  |  |  |  |  |  |  |
| **Up to high school** |  | 46 (57.1) | 47.8 | 66 | 26 (63.3) | 48.9 | 75.7 | 49 (59.4) | 50.2 | 68 |
| **Higher education** |  | 37 (42.9) | 34 | 52.2 | 17 (36.7) | 24.3 | 51.1 | 40 (40.6) | 32 | 49.8 |
| **Belonging to EAP** | <.001 | 57 (68.7) | 59 | 77 | 32 (68.6) | 53.6 | 80.5 | 54 (59.3) | 49.4 | 68.6 |
| **Mean age** | <.001 | 34.5 | 31.5 | 37.6 | 36.3 | 31.1 | 41.5 | 28.4 | 25.8 | 31.1 |
| **Mean number of children** | <.001 | 0.59 | 0.33 | 0.86 | 0.99 | 0.56 | 1.41 | 0.64 | 0.40 | 0.88 |
| **Variable** | ***p*** | **Group (n = 5858)** | | **(Continued).** | |  |  |  |  |  |
|  |  | **Trans man, n = 20 (0.34%, CI 95% = 0.22 to 0.52)** | | | **Trans woman, n = 20 (0.34%, CI 95% = 0.22 to 0.53)** | | | **Non-binary, n = 71 (1.18%, CI 95% = 0.94 to 1.49)** | | |
|  |  | **n (%)** | **CI 95%** | | **n (%)** | **CI 95%** | | **n (%)** | **CI 95%** | |
| **Social class** | <.001 |  |  |  |  |  |  |  |  |  |
| **D/E** |  | 10 (53.2) | 32.6 | 72.8 | 6 (36) | 17.6 | 59.7 | 20 (30.2) | 20.4 | 42.2 |
| **C** |  | 7 (34.3) | 17.3 | 56.5 | 8 (42) | 22.6 | 64.1 | 32 (48.8) | 37.5 | 60.3 |
| **A/B** |  | 3 (12.5) | 4.0 | 32.6 | 6 (22.1) | 10.5 | 40.6 | 19 (21) | 13.6 | 31.0 |
| **Urbanity** | <.001 |  |  |  |  |  |  |  |  |  |
| **Countryside** |  | 6 (28.1) | 13.2 | 50.3 | 7 (32.4) | 15.9 | 54.9 | 19 (24.9) | 16.3 | 36.1 |
| **Metropolitan area** |  | 14 (71.9) | 49.7 | 86.8 | 13 (67.6) | 45.1 | 84.1 | 52 (75.1) | 63.9 | 83.7 |
| **Relationship status** | <.001 |  |  |  |  |  |  |  |  |  |
| **Not in a relationship** |  | 10 (46.7) | 27.1 | 67.3 | 16 (82.9) | 61 | 93.8 | 46 (67) | 55.4 | 76.9 |
| **In a Relationship** |  | 10 (53.3) | 32.7 | 72.9 | 4 (17.1) | 6.2 | 39 | 25 (33) | 23.1 | 44.6 |
| **Subregion** | <.07 |  |  |  |  |  |  |  |  |  |
| **Southeast** |  | 7 (37.8) | 20.4 | 59.2 | 11 (56.2) | 34.6 | 75.7 | 30 (45.6) | 34.4 | 57.3 |
| **South** |  | 3 (11.4) | 3.7 | 30.5 | 3 (13.4) | 4.3 | 34.8 | 12 (14.2) | 8.5 | 22.8 |
| **Northeast** |  | 8 (38.9) | 20.9 | 60.5 | 3 (14.9) | 4.8 | 37.6 | 21 (29.3) | 19.9 | 40.9 |
| **Central west** |  | 0 (0) | 0 | 0 | 1 (5) | 0.7 | 28.1 | 5 (6.5) | 2.7 | 15 |
| **North** |  | 2 (11.8) | 3 | 36.5 | 2 (10.5) | 2.3 | 36.8 | 3 (4.4) | 1.4 | 12.9 |
| **Education** | <.001 |  |  |  |  |  |  |  |  |  |
| **Up to high school** |  | 19 (96.1) | 76.6 | 99.5 | 16 (85.8) | 70.7 | 93.9 | 57 (82.7) | 73.2 | 89.4 |
| **Higher education** |  | 1 (3.9) | 0.5 | 23.4 | 4 (14.2) | 6.1 | 29.3 | 14 (17.3) | 10.6 | 26.8 |
| **Belonging to EAP** | <.001 | 8 (37) | 19.6 | 58.6 | 13 (65.1) | 42.2 | 82.7 | 47 (65.6) | 53.8 | 75.6 |
| **Mean age** | <.001 | 35.3 | 30.0 | 40.5 | 30.4 | 23.8 | 37.0 | 42.4 | 38.4 | 46.4 |
| **Mean number of children** | <.001 | 1.72 | 1.11 | 2.32 | 0.44 | -0.09 | 0.97 | 1.52 | 1.18 | 1.85 |
| **Variable** | ***p*** | **Group (n = 5858)** | | **(Continued).** | |  |  |  |  |  |
|  |  | **Asexual man, n = 22 (0.37%, CI 95% = 0.25 to 0.55)** | | | **Asexual woman, n = 303 (5.39%, CI 95% = 4.88 to 5.95)** | | |  |  |  |
|  |  | **n (%)** | **CI 95%** | | **n (%)** | **CI 95%** | |  |  |  |
| **Social class** | <.001 |  |  |  |  |  |  |  |  |  |
| **D/E** |  | 7 (35.5) | 18.5 | 57.2 | 131 (45.7) | 40.3 | 51.2 |  |  |  |
| **C** |  | 8 (36.3) | 19.5 | 57.3 | 131 (44) | 38.7 | 49.5 |  |  |  |
| **A/B** |  | 7 (28.2) | 15.0 | 46.6 | 41 (10.3) | 7.8 | 13.4 |  |  |  |
| **Urbanity** | <.001 |  |  |  |  |  |  |  |  |  |
| **Countryside** |  | 6 (24) | 11.0 | 44.7 | 71 (21.3) | 17.6 | 25.5 |  |  |  |
| **Metropolitan area** |  | 16 (76) | 55.3 | 89.0 | 232 (78.7) | 74.5 | 82.4 |  |  |  |
| **Relationship status** | <.001 |  |  |  |  |  |  |  |  |  |
| **Not in a relationship** |  | 16 (73.7) | 53.2 | 87.3 | 228 (75.8) | 70.9 | 80.2 |  |  |  |
| **In a Relationship** |  | 6 (26.3) | 12.7 | 46.8 | 75 (24.2) | 19.8 | 29.1 |  |  |  |
| **Subregion** | <.07 |  |  |  |  |  |  |  |  |  |
| **Southeast** |  | 11 (52.4) | 32.9 | 71.2 | 143 (48.8) | 43.9 | 53.8 |  |  |  |
| **South** |  | 5 (22.1) | 10.4 | 41.1 | 42 (13.2) | 10.2 | 16.8 |  |  |  |
| **Northeast** |  | 5 (22.9) | 9.9 | 44.4 | 83 (25.4) | 21.4 | 29.8 |  |  |  |
| **Central west** |  | 1 (2.6) | 0.4 | 16.5 | 18 (6.5) | 4.5 | 9.4 |  |  |  |
| **North** |  | 0 (0) | 0 | 0 | 17 (6.1) | 3.9 | 9.5 |  |  |  |
| **Education** | <.001 |  |  |  |  |  |  |  |  |  |
| **Up to high school** |  | 15 (71.3) | 51.9 | 85.2 | 272 (90.8) | 88.1 | 92.9 |  |  |  |
| **Higher education** |  | 7 (28.7) | 14.8 | 48.1 | 31 (9.2) | 7.1 | 11.9 |  |  |  |
| **Belonging to EAP** | <.001 | 9 (40.2) | 22.6 | 60.8 | 99 (32.6) | 27.6 | 38 |  |  |  |
| **Mean age** | <.001 | 54 | 45.9 | 62.1 | 59.7 | 58.4 | 61.0 |  |  |  |
| **Mean number of children** | <.001 | 1.21 | 0.72 | 1.7 | 2.33 | 2.18 | 2.49 |  |  |  |

Note: CI = confidence interval. EAP = economically active population. Percentages, means, and CIs were design-adjusted.

Supplementary table 3. Association between reporting violence and variable Group.

| **Type of violence** | ***p*** | **Group (n = 5854)** | |  |  |  |  |  |  |  |
| --- | --- | --- | --- | --- | --- | --- | --- | --- | --- | --- |
|  |  | **Heterosexual cis man, n = 2589 (42.5%, CI 95% = 42.01 to 43.00)** | | | **Heterosexual cis woman, n = 2563 (43.02%, CI 95% = 42.3 to 43.73)** | | | **Lesbian, n = 55 (0.93%, CI 95% = 0.72 to 1.21)** | | |
|  |  | **n (%)** | **CI 95%** | | **n (%)** | **CI 95%** | | **n (%)** | **CI 95%** | |
| **Psychological** | <.001 | 690 (26.48) | 24.88 | 28.15 | 747 (29.47) | 27.8 | 31.2 | 16 (27.59) | 17.68 | 40.31 |
| **Physical** | <.001 | 727 (28.13) | 26.5 | 29.82 | 517 (20.79) | 19.28 | 22.39 | 11 (20.34) | 11.57 | 33.26 |
| **Verbal** | <.001 | 1377 (52.96) | 51.12 | 54.8 | 1264 (49.65) | 47.81 | 51.5 | 36 (63.25) | 49.61 | 75.05 |
| **Sexual** | <.001 | 50 (1.89) | 1.45 | 2.45 | 198 (7.59) | 6.67 | 8.63 | 7 (11.49) | 5.45 | 22.63 |
| **Type of violence** | ***p*** | **Group (n = 5854)** | | **(Continued).** | |  |  |  |  |  |
|  |  | **Gay, n = 83 (1.37%, CI 95% = 1.13 to 1.65)** | | | **Bisexual man, n = 43 (0.7%, CI 95% = 0.53 to 0.94)** | | | **Bisexual woman, n = 89 (1.42%, CI 95% = 1.17 to 1.72)** | | |
|  |  | **n (%)** | **CI 95%** | | **n (%)** | **CI 95%** | | **n (%)** | **CI 95%** | |
| **Psychological** | <.001 | 31 (37.5) | 28.59 | 47.35 | 16 (39.2) | 26.17 | 53.97 | 44 (49.18) | 39.31 | 59.12 |
| **Physical** | <.001 | 21 (25.2) | 17.5 | 34.85 | 13 (32.53) | 20.79 | 46.98 | 29 (33.11) | 24.4 | 43.15 |
| **Verbal** | <.001 | 40 (47.99) | 38.23 | 57.9 | 27 (63.79) | 48.57 | 76.66 | 60 (66.55) | 56.61 | 75.22 |
| **Sexual** | <.001 | 8 (10.41) | 5.29 | 19.48 | 5 (12.97) | 6.06 | 25.6 | 21 (22.74) | 15.44 | 32.18 |
| **Type of violence** | ***p*** | **Group (n = 5854)** | | **(Continued).** | |  |  |  |  |  |
|  |  | **Trans man, n = 20 (0.34%, CI 95% = 0.22 to 0.52)** | | | **Trans woman, n = 20 (0.34%, CI 95% = 0.22 to 0.53)** | | | **Non-binary, n = 71 (1.18%, CI 95% = 0.94 to 1.49)** | | |
|  |  | **n (%)** | **CI 95%** | | **n (%)** | **CI 95%** | | **n (%)** | **CI 95%** | |
| **Psychological** | <.001 | 12 (58.35) | 36.78 | 77.14 | 12 (57.39) | 35.19 | 76.96 | 28 (39.76) | 28.97 | 51.64 |
| **Physical** | <.001 | 6 (34.34) | 17.65 | 56.05 | 9 (43.05) | 23.53 | 65.01 | 26 (38.11) | 27.38 | 50.15 |
| **Verbal** | <.001 | 13 (66.91) | 44.91 | 83.38 | 14 (67.06) | 43.44 | 84.37 | 50 (70.33) | 58.61 | 79.88 |
| **Sexual** | <.001 | 8 (39.03) | 21.54 | 59.87 | 6 (29.93) | 13.73 | 53.41 | 16 (22.88) | 14.47 | 34.22 |
| **Type of violence** | ***p*** | **Group (n = 5854)** | | **(Continued).** | |  |  |  |  |  |
|  |  | **Asexual man, n = 22 (0.37%, CI 95% = 0.25 to 0.55)** | | | **Asexual woman, n = 303 (5.39%, CI 95% = 4.88 to 5.95)** | | |  |  |  |
|  |  | **n (%)** | **CI 95%** | | **n (%)** | **CI 95%** | |  |  |  |
| **Psychological** | <.001 | 4 (19.51) | 7.6 | 41.66 | 92 (31.04) | 26.12 | 36.43 |  |  |  |
| **Physical** | <.001 | 3 (13.17) | 4.12 | 34.85 | 81 (26.77) | 22.16 | 31.94 |  |  |  |
| **Verbal** | <.001 | 11 (50.58) | 31.28 | 69.71 | 153 (51.52) | 46.08 | 56.92 |  |  |  |
| **Sexual** | <.001 | 0 (0) | 0 | 0 | 28 (9.13) | 6.5 | 12.68 |  |  |  |

Note: CI = confidence interval. Percentages, means, and CIs were design-adjusted.

STROBE Statement—Checklist of items that should be included in reports of ***cross-sectional studies***

|  | Item No | Recommendation | Page No |
| --- | --- | --- | --- |
| Title and abstract | 1 | (*a*) Indicate the study’s design with a commonly used term in the title or the abstract | 1 |
|  |  | (*b*) Provide in the abstract an informative and balanced summary of what was done and what was found | 2 |
| Introduction | | | |
| Background/rationale | 2 | Explain the scientific background and rationale for the investigation being reported | 3 |
| Objectives | 3 | State specific objectives, including any prespecified hypotheses | 3 |
| Methods | | | |
| Study design | 4 | Present key elements of study design early in the paper | 3 |
| Setting | 5 | Describe the setting, locations, and relevant dates, including periods of recruitment, exposure, follow-up, and data collection | 3 |
| Participants | 6 | (*a*) Give the eligibility criteria, and the sources and methods of selection of participants | 3 |
| Variables | 7 | Clearly define all outcomes, exposures, predictors, potential confounders, and effect modifiers. Give diagnostic criteria, if applicable | 4 |
| Data sources/ measurement | 8* | For each variable of interest, give sources of data and details of methods of assessment (measurement). Describe comparability of assessment methods if there is more than one group | 4 |
| Bias | 9 | Describe any efforts to address potential sources of bias | 5 |
| Study size | 10 | Explain how the study size was arrived at | 3 |
| Quantitative variables | 11 | Explain how quantitative variables were handled in the analyses. If applicable, describe which groupings were chosen and why | 4 |
| Statistical methods | 12 | (*a*) Describe all statistical methods, including those used to control for confounding | 5 |
|  |  | (*b*) Describe any methods used to examine subgroups and interactions | 5 |
|  |  | (*c*) Explain how missing data were addressed | 5 |
|  |  | (*d*) If applicable, describe analytical methods taking account of sampling strategy | 5 |
|  |  | (*e*) Describe any sensitivity analyses | N/A |
| Results | | | |
| Participants | 13* | (a) Report numbers of individuals at each stage of study—eg numbers potentially eligible, examined for eligibility, confirmed eligible, included in the study, completing follow-up, and analysed | 6 |
|  |  | (b) Give reasons for non-participation at each stage | 6 |
|  |  | (c) Consider use of a flow diagram | NA |
| Descriptive data | 14* | (a) Give characteristics of study participants (eg demographic, clinical, social) and information on exposures and potential confounders | 6 |
|  |  | (b) Indicate number of participants with missing data for each variable of interest | NA |
| Outcome data | 15* | Report numbers of outcome events or summary measures | 6 |
| Main results | 16 | (*a*) Give unadjusted estimates and, if applicable, confounder-adjusted estimates and their precision (eg, 95% confidence interval). Make clear which confounders were adjusted for and why they were included | 6 |
|  |  | (*b*) Report category boundaries when continuous variables were categorized | 6 |
|  |  | (*c*) If relevant, consider translating estimates of relative risk into absolute risk for a meaningful time period | 6 |
| Other analyses | 17 | Report other analyses done—eg analyses of subgroups and interactions, and sensitivity analyses | NA |
| Discussion | | | |
| Key results | 18 | Summarise key results with reference to study objectives | 7 |
| Limitations | 19 | Discuss limitations of the study, taking into account sources of potential bias or imprecision. Discuss both direction and magnitude of any potential bias | 10 |
| Interpretation | 20 | Give a cautious overall interpretation of results considering objectives, limitations, multiplicity of analyses, results from similar studies, and other relevant evidence | 10 |
| Generalisability | 21 | Discuss the generalisability (external validity) of the study results | 10 |
| Other information | | | |
| Funding | 22 | Give the source of funding and the role of the funders for the present study and, if applicable, for the original study on which the present article is based | NA |
